# Instrumental resolution as a function of scattering angle and wavelength as exemplified for the POWGEN instrument

**DOI:** 10.1107/S1600576717005398

**Published:** 2017-05-25

**Authors:** Philipp Jacobs, Andreas Houben, Werner Schweika, Andrei L. Tchougréeff, Richard Dronskowski

**Affiliations:** a Institute of Inorganic Chemistry, RWTH Aachen University, D-52056 Aachen, Germany; b European Spallation Source ESS, SE-22100 Lund, Sweden; c Jülich Center for Neutron Science and Peter Grünberg Institute PGI, Forschungszentrum Jülich, D-52425 Jülich, Germany; d A. N. Frumkin Institute of Physical Chemistry and Electrochemistry of Russian Academy of Science, and Poncelet Laboratory of Mathematics in Interaction with Physics and Informatics, Independent University of Moscow, Moscow Center for Continuous Mathematical Education, Bolshoi Vlasevsky Pereulok 11, 119002 Moscow, Russian Federation

**Keywords:** instrumental resolution, POWGEN, time of flight, neutron diffraction, angular- and wavelength-dispersive Rietveld refinement, powder methods

## Abstract

The method of angular- and wavelength-dispersive (*e.g.* two-dimensional) Rietveld refinement is a new and emerging tool for the analysis of neutron diffraction data measured at time-of-flight instruments with large area detectors. Following the approach for one-dimensional refinements (using either scattering angle or time of flight), the first step at each beam time cycle is the calibration of the instrument including the determination of instrumental contributions to the peak shape variation to be expected for diffraction patterns measured by the users. The aim of this work is to provide the users with calibration files and – for the later Rietveld refinement of the measured data – with an instrumental resolution file (IRF). This article will elaborate on the necessary steps to generate such an IRF for the angular- and wavelength-dispersive case, exemplified for the POWGEN instrument. A dataset measured on a standard diamond sample is used to extract the profile function in the two-dimensional case. It is found that the variation of reflection width with 2θ and λ can be expressed by the standard equation used for evaluating the instrumental resolution, which yields a substantially more fundamental approach to the parameterization of the instrumental contribution to the peak shape. Geometrical considerations of the POWGEN instrument and sample effects lead to values for Δθ, Δ*t* and Δ*L* that yield a very good match to the extracted FWHM values. In a final step the refinement results are compared with the one-dimensional, *i.e.* diffraction-focused, case.

## Abstract 2

A fundamental description of the instrument resolution file is elaborated for the angular- and wavelength-dispersive cases of Rietveld refinement, exemplified for the POWGEN instrument. It is shown how to refine the necessary profile function parameters from a dataset measured on a diamond reference sample. The analysis is performed in a two-dimensional refinement space based on the convenient variables *d* and *d*_⊥_.

## 1. Introduction   

Since its invention in the late 1960s the Rietveld method (Rietveld, 1969 ▸) has been widely used for the investigation of structural and – using neutron powder diffraction – even magnetic properties of powdered polycrystalline samples. One of the prerequisites for the analytical description of total integrated intensities by a least-squares fit of the full diffraction profile is the appropriate description of the individual peak shapes. When the Rietveld method was first introduced mainly Gaussian distributions were considered, but over time more and more complex peak shapes have been added to the pool of usable peak profiles, culminating in highly sophisticated peak shape functions such as pseudo-Voigt with back-to-back-exponentials (Von Dreele *et al.*, 1982 ▸) as used at modern time-of-flight (TOF) diffractometers.

When trying to properly describe the peak shape, it immediately becomes apparent that a plethora of different effects are contributing. These can be divided into instrumental effects arising from the neutron source as well as the layout of the instrument – causing a change in instrumental resolution with the scattering variables – and the intrinsically more interesting sample effects (*e.g.* crystal structure, particle size, micro-strain or texture effects). In general, a so-called instrumental resolution file (IRF) is provided to the user of a modern TOF instrument, comprising the basic parameters for a description of the source/instrument contribution to the measured peak shapes. These parameters should remain fixed during the refinement procedure such that deviations from the observed peak shape have to be addressed by additional sample-dependent terms.

Modern powder diffractometers with large area detectors at advanced neutron spallation sources typically operate in TOF mode, initially resulting in angular- and energy-dispersive datasets. The current approach at existing TOF instruments is to reduce, transform and integrate the accumulated data to the well known one-dimensional diffraction patterns (Schäfer *et al.*, 1992 ▸; http://www.mantidproject.org) that can be routinely treated using standard Rietveld refinement packages (Bruker, 2005 ▸; Toby & Von Dreele, 2013 ▸; Larson & Von Dreele, 1994 ▸; Lutterotti *et al.*, 1999 ▸; Rodriguez-Carvajal, 1993 ▸; Petříček *et al.*, 2014 ▸). This procedure has the advantage of providing readily refinable diffraction datasets that are relatively small in size and thus allow for quick calculation times. A significant amount of the originally available information is lost, however, and cannot be exploited. Furthermore, regions of varying resolution are integrated for single reflections, which can ultimately lead to a complex variation of the peak shape in a single diffraction pattern.

It would therefore be highly favorable to avoid these data-treatment steps and use the neutron diffraction data in its original two-dimensional (*i.e*. intensity as a function of 2θ and TOF/λ) form. Up to now, only very few scientific publications could be found that are at least related to this important topic (Schäfer *et al.*, 1992 ▸; Wang *et al.*, 2004 ▸).

In order to improve the data analysis capabilities for powder diffraction, we have recently devised (Jacobs *et al.*, 2015 ▸) an alternative approach using the diffraction data in their pristine form and implementing a two-dimensional description of the peak shape variation. In this study we build upon the foundations laid out in the quoted paper and not only refine but also advance our approach. This is achieved by closely examining the dataset of a standard diamond sample measured at the POWGEN instrument, which serves as one suitable reference for the instrument resolution function (Huq *et al.*, 2010 ▸). POWGEN is a medium- to high-resolution instrument featuring a large area detector designed and built to optimally match the angular resolution Δθcotθ to the almost constant relative time resolution 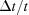 (Huq *et al.*, 2010 ▸), resulting in an almost constant Δ*d*/*d* within the total frame of a measurement. Thus, at first glance, the POWGEN instrument might not seem to be the best choice for such an investigation, but we will show that even for an instrument optimized for constant resolution one may gain a significant benefit in the characterization of the instrumental resolution by the multi-dimensional approach, mainly because, in addition to the width, the shape also changes with 2θ and λ. Additionally, most other time-of-flight powder diffractometers have less homogeneous resolution functions: see for example the instruments WISH, POLARIS, HRPD at ISIS and Super-HRPD at JPARC. Currently, the valid approaches in data analysis are more or less workarounds, which either ignore most of the data and focus on the region of interest (*e.g.* backscattering for high resolution) or group data according to different detector banks for a multi-pattern Rietveld refinement process. The ability to deal with varying resolution functions also allows for a simpler and more cost-effective detector design, as seen for future instruments such as POWTEX (Conrad *et al.*, 2008 ▸; Houben *et al.*, 2012 ▸) and DREAM (Schweika *et al.*, 2016 ▸). In general, all these instruments will hugely benefit from the two-dimensional approach.

The ultimate goal will thus be to illustrate the necessary steps for a full parameterization of the instrumental resolution function and, on this basis, provide an IRF for the angular- and wavelength-dispersive case.

## 2. Neutron powder diffraction data   

A standard diamond sample (see Table 1 ▸ for crystallographic data) was measured on the POWGEN instrument (SNS, Oak Ridge National Laboratory) for 6.5 h at room temperature using the instrument setup according to bank 1 (λ_Center_ = 0.533 Å). This measurement was carried out by the instrument team of POWGEN and the data were kindly given to us by Ashfia Huq, lead scientist at POWGEN. The total number of neutron events in this measurement was approximately 150 million in a 2θ range between 17.5 and 150° and a λ range between close to 0 and 1.101 Å. For correction and normalization purposes, measurements of a vanadium sample and the empty sample can were provided, with each dataset containing approximately 72 million and 3.7 million neutron events, respectively. The diamond, vanadium and background datasets were treated as reported previously (Jacobs *et al.*, 2015 ▸). Owing to the high ratio between coherent and incoherent scattering cross sections of carbon (5.55:0.001 b; 1 b = 100 fm^2^; Dianoux & Lander, 2003 ▸) only a small portion of the neutrons add to the background, while the majority of the detected neutrons contribute to the reflections. Hence, the signal-to-noise ratio in this dataset is (slightly) better than that in the CuNCN sample used in our previous study, which was also measured at POWGEN for approximately 7.5 h.

## 3. Parameterization of the instrument (instrumental resolution function)   

**Figure 1:**
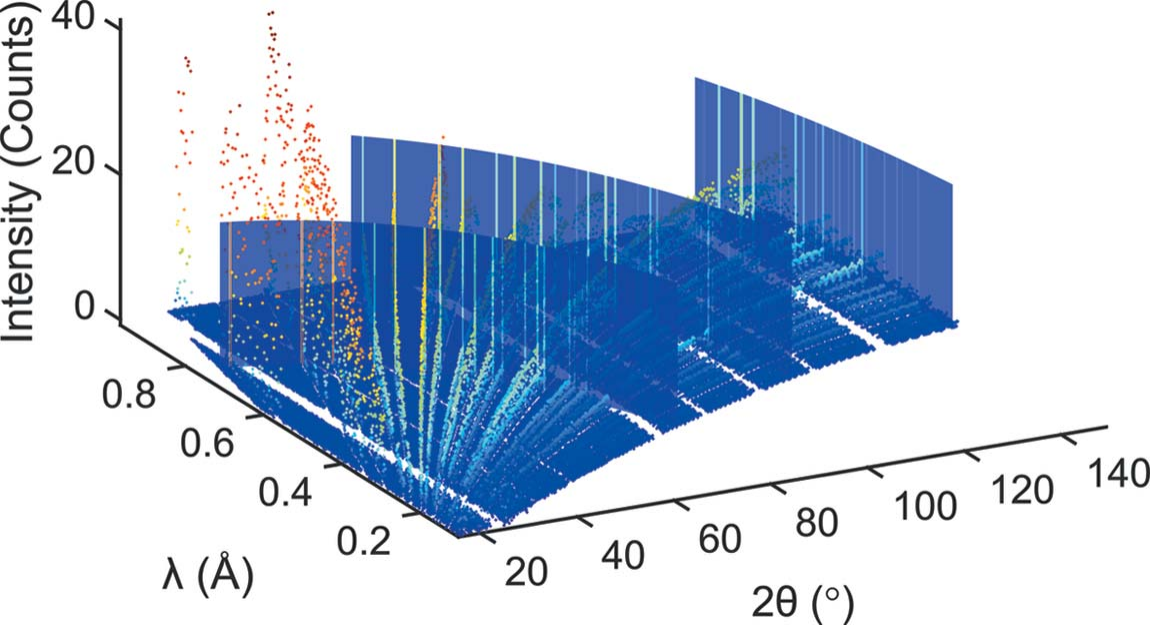
Three-dimensional plot of the neutron powder diffraction pattern of diamond. Opaque surfaces represent the intensity distribution along the orthonormal cuts at *d*_⊥_ = 0.75, 1.12 and 1.68 Å.

**Figure 2:**
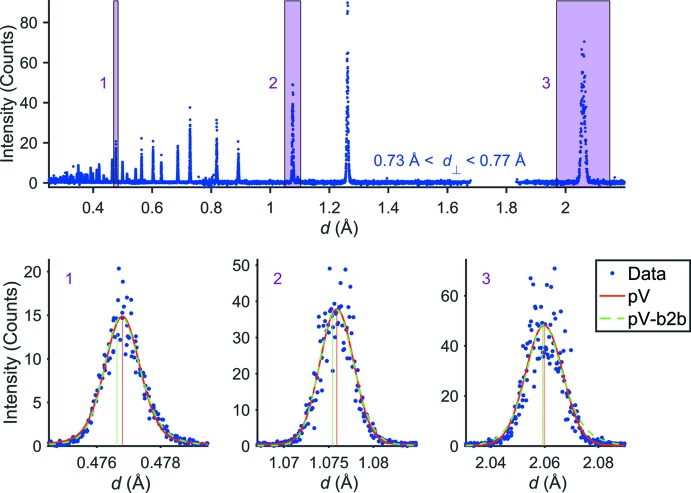
Orthogonal cut with data points in the range 0.73 < *d*_⊥_ < 0.77 Å (upper part). Comparison of the fits using either a pseudo-Voigt with back-to-back exponentials (red solid line) or a standard pseudo-Voigt function (green dashed line) for reflections 111 (3), 311 (2) and 642 (1).

For a full two-dimensional parameterization of the instrumental resolution function of POWGEN and, ultimately, to derive an instrumental resolution file, we used a high-neutron-count dataset measured on a standard diamond sample. This standard sample is normally used to derive the calibration file, for example the *d* offsets for each detector pixel. The accurate determination of *d* offsets necessitates a rather high overall neutron count, which also benefits the angular- and wavelength-dispersive refinement approach. As has already been pointed out (Jacobs *et al.*, 2015 ▸) the first step towards a full parameterization of the instrumental resolution and thus a full description of the reflection shape variation consists of extracting the shape-defining parameters out of a measured dataset taken from a ‘standard’ sample. Thereby one assumes the final diffraction pattern – and to an even greater extent the profile shape of each reflection – to be almost free of sample effects. As has been shown before (Jacobs *et al.*, 2015 ▸) the best approach to extract these parameters is to use slices of small data intervals Δ*d*_⊥_ which are distributed around the orthonormal curve given by 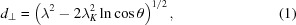
where λ is the wavelength and θ denotes the Bragg angle. Here, we have chosen the (arbitrary) constant 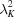 = 1 Å^2^, which defines specific and appropriate orthogonal coordinates *d* and *d*_⊥._. Three of these orthonormal curves together with the three-dimensional representation of the dataset are depicted in Fig. 1 ▸. The data points in the interval {*d*_⊥_ ∈ R | 0.73 ≤ *d*_⊥_ ≤ 0.77 Å}, corresponding to the leftmost slice in Fig. 1 ▸, are plotted in Fig. 2 ▸ (upper part), together with three highlighted reflections (lower part). Typically, the peak profile for refining diffraction patterns obtained at the POWGEN instrument is approximated using a pseudo-Voigt function with back-to-back exponentials (pV-b2b). The same notation was used to fit the reflections shown in the lower part of Fig. 2 ▸ (green dashed line). In addition, each peak was also fitted using a standard pseudo-Voigt (pV) function (red solid line). By comparing both fit results it becomes apparent that using the standard pV notation yields comparable results for the full width at half-maximum (FWHM), while even simplifying the refinement owing to the absence of the rise-and-decay parameters of the back-to-back exponentials. The *d* value, in contrast, is different for the two peak shapes; the pV function with back-to-back exponentials usually shifts to lower *d* values as was already elaborated on by Von Dreele *et al.* (1982 ▸). For the determination of the peak width we can therefore safely resort to the use of a standard pseudo-Voigt peak profile such that only two parameters, the FWHM (denoted as *H* herein) and the Lorentzian mixing parameter (η), must be extracted from a sufficiently large number of points in the diffraction pattern. The alternative description using the Thompson–Cox–Hastings (Thompson *et al.*, 1987 ▸) notation (*e.g.* unique FWHM parameters for the Gaussian and Lorentzian components) in principle opens up a somewhat more physically meaningful handling of the profile in the one-dimensional case. This is because sample-related effects can be more strictly assigned to either the Gaussian or the Lorentzian part, depending on their own type of distribution. For the present study we will nevertheless resort to a parameterization of the complete FWHM as a function of the scattering angle and the wavelength.

When using *d*_⊥_ slices the analysis of the reflection shape is done by applying an individual FWHM but a common η value to each reflection contained in a single *d*_⊥_ slice. Fitting of the pV function to the data points of a single reflection is done using the intensity *I* as a function of *d* spacing: 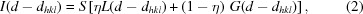
where *L*(*d* − *d_hkl_*) is the Lorentzian part given by 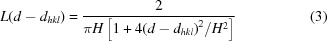
and *G*(*d* − *d*_*hkl*_) is the Gaussian part, 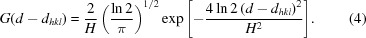
In these equations *S* is a scaling factor, η is the Lorentzian mixing parameter, *H* denotes the FWHM of the reflection *hkl*, *d* is the *d* value of each data point and *d_hkl_* is the central *d* value of the reflection around which the intensity is calculated. This has the distinct advantage that no additional parameters are needed to convert time of flight to *d* spacing as is commonly needed for today’s refinement programs.

**Figure 3:**
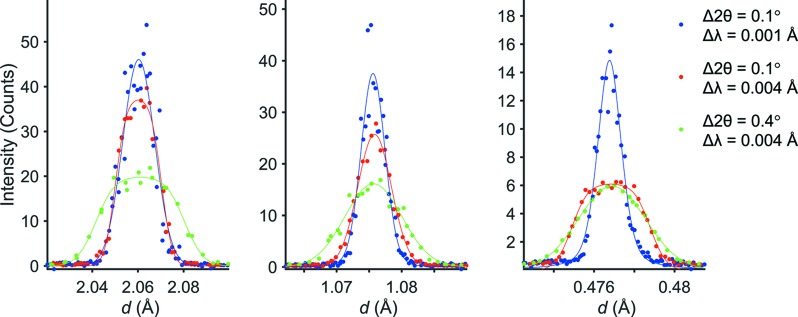
Influence of bin sizes on the FWHM (and overall shape) of the reflections 111 (left), 311 (middle) and 642 (right), considering data points around a central *d*_⊥_ value of 0.75 Å. Solid lines are just a guide to the eye.

When evaluating the results of the extracted *H* values for the datasets binned in 2θ and λ, it becomes apparent that the bin size of either has an immediate impact. In Table 2 ▸ we provide a comparison of *H* values for reflections at *d* = 0.477, 1.076 and 2.059 Å using data points in a slice with {*d*_⊥_ ∈ R | 0.73 ≤ *d*_⊥_ ≤ 0.77 Å} and different bin sizes. Apparently, the FWHM strongly depends on the chosen bin size and varies significantly with the slope of the Bragg curves for each reflection. Reflections at higher *d* values (steeper reflections; to the left) are highly influenced by too broad a binning in 2θ (FWHM increases by ∼300%), while the binning of λ is only of minor importance. For lower *d* values (flat slope; horizontal to the right) the binning of λ has the highest impact (increase of ∼200% in FWHM), whereas the 2θ binning has only a small influence. Reflections at medium *d* values are influenced by both bin sizes and show an increase of FWHM of ∼300%. Fig. 3 ▸ shows this by comparing different binning schemes for the three reflections. Apart from the FWHM, it becomes obvious that with larger bin sizes the pV function is not an adequate description of the intensity distribution anymore, but the peak shapes become more and more ‘top hat’ like (see Fig. 3 ▸, right). If the binning is too coarse, the peak shape is clearly dominated by binning effects, which should be avoided. Additionally, the density of data points is smaller for larger bin values so that especially at low *d* spacing there are an insufficient number of data points describing each reflection. On this account the *d*_⊥_ range for selecting the data points for each binning method shown in Fig. 3 ▸ had to be individually adjusted for each reflection to give at least ten contributing data points per FWHM. Note that we will introduce a mechanism to ensure that this bin size requirement per FWHM is globally met for the entire pattern. For the moment, let us assume that the change of the FWHM is not affected by the bin sizes, *i.e.* the number of points/FWHM is sufficient.

**Figure 4:**
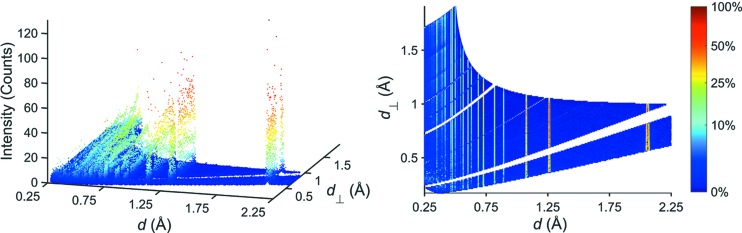
Two-dimensional (left) and quasi-two-dimensional (right) representation of the neutron powder diffraction data of diamond binned in *d* and *d*_⊥_ using bin sizes of Δ = 0.0008 and Δ*d*_⊥_ = 0.00872 Å.

Fortunately, there is an alternative to the 2θ and λ binning approach that allows an easier treatment of the resolution change. As was already laid out in our previous paper, it is better to use *d* and *d*_⊥_ as new coordinates which span an orthogonal coordinate system where all reflections are represented by straight lines (see Fig. 4 ▸), while the orthogonal cut of the reflection is a horizontal line. This can be done simply by using equation (1) and the Bragg equation
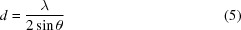
to convert the 2θ and λ values of each detected neutron event to *d* and *d*_⊥_ and binning all neutron events in this tailor-made coordinate system. Owing to the constant monitoring of the two independent variables, an easy transformation between the two coordinate systems is always possible, including during the refinement. Replacing θ in equation (1) by 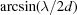 and solving the equation for λ leads to the following equation: 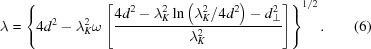
Herein ω is the Wright omega function (Wright, 1959 ▸; Corless & Jeffrey, 2002 ▸), which is defined in terms of the Lambert W function (Lambert, 1758 ▸; Corless *et al.*, 1996 ▸). Accessing different coordinate systems during the refinement will allow for corrections as regards extinction, absorption and other effects (Sabine *et al.*, 1988 ▸) on the intensity data in their natural dependency. Furthermore, this in principle also allows us to reuse or even combine existing parameterizations in 2θ and/or λ with a *d*/*d*_⊥_ binning.

For a first approach the binning in *d* can be done (as is usual) using a quasi-logarithmic binning scheme for POWGEN with bin boundaries calculated by 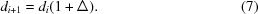
For the standard data reduction procedure at the POWGEN instrument a default value of Δ = 0.0008 is chosen. We therefore used values in the same range for the datasets analyzed in this study, whereas for *d*_⊥_ we chose a linear binning scheme. To check if the bin size in *d*_⊥_ and especially in *d* has a major impact on the extracted *H* values and the shape of the reflections, we investigated final datasets with a linear binning in *d*_⊥_ giving 40, 80 and 200 *d*_⊥_ slices as well as logarithmic binning in *d* using Δ = 0.0004, 0.0008 and 0.0016, respectively. The data range of each dataset was limited to 0.25 ≤ *d* ≤ 0.97 Å and 0.17 ≤ *d*_⊥_ ≤ 1.91 Å. A comparison of the resulting *H* values for the same reflections used earlier employing a slice at *d*_⊥_ = 0.75 Å is given in Table 3 ▸. It is obvious that the FWHM is considerably less influenced by the bin sizes of *d* and *d*_⊥_, even for a very large value of Δ = 0.0016. If compared with the results of the 2θ and λ binning, only the smallest bin sizes of Δ2θ = 0.05° and Δλ = 0.0005 Å conform to the results of the *d* and *d*_⊥_ binning. Nevertheless, the number of data points per FWHM changes for each reflection, with the one at the lowest *d* value having significantly fewer data points per FWHM than the reflections at higher *d* values. Thus, a new resolution-adapted binning scheme is presented, where for each *d*_⊥_ bin the *d* bin limits are calculated according to 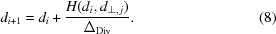
Here, *H*(*d_i_*, *d*_⊥,*j*_) is the FWHM calculated using the central value of the *j*th *d*_⊥_ bin and the upper *d* limit of the previous *d* bin. Δ_Div_ is simply a divisor that represents the desired number of data points per FWHM. Using this binning scheme, a homogeneous distribution of data points can be achieved, which yields between five and ten data points per FWHM for each reflection, as has been suggested by McCusker *et al.* (1999 ▸). The resulting FWHMs of this binning scheme are summarized in Table 4 ▸. Using the resolution-adapted binning scheme, the number of data points per FWHM is constant throughout, with no significant influence on the FWHM. Hence, it seems highly recommendable to use the resolution-adapted *d* and *d*_⊥_ binning scheme for analyzing angular- and wavelength-dispersive datasets. In the following we therefore concentrate on the dataset using 40 *d*_⊥_ slices and approximately ten data points per FWHM as this is an excellent compromise between computational effort, good counting statistics and reasonable data-point density. The one-dimensional approach is the limiting case for a single *d*_⊥_ bin.

Once any influence on the peak profile stemming from the binning scheme can be excluded, one may derive an analytical description for the distribution of *H* values. Ideally, one should try to mirror the underlying physical principles contributing to the intensity distribution of the reflections. Typically, the resolution and thus the FWHM of each reflection is approximated using the following equation: 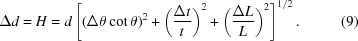
Herein Δθ, Δ*t* and Δ*L* are the uncertainties in scattering angle, TOF and total flight path, respectively. θ denotes the scattering angle, *t* is the time of flight and *L* is the total flight path from the source to the detector. To derive meaningful values for all three contributions one needs to take a closer look at the instrument, sample and source properties.

The angular resolution is given by the layout of the detector (Δθ_Detector_), the divergence of the neutron beam (Δθ_Divergence_), and the sample size and shape (Δθ_Sample_): 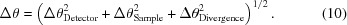
Since POWGEN’s detectors are mainly arranged around an azimuthal angle of φ = 0, we are mainly concerned with the in-plane probability distribution of the neutrons scattered from the sample. The uncertainty in scattering angle caused by the detector is therefore given by the horizontal extent (= width) of each detector pixel (*w*_Detec_) and the distance *L*_2_ of the detector pixel from the sample (‘secondary flight path’): 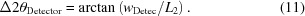

*w*_Detec_ of each detector pixel is the FWHM of a continuous uniform distribution with size equal to the horizontal extent of the detector pixel. For a detector pixel width of 5 mm the uniform distribution width is calculated to be *w*_Detec_ = 3.39 mm.

The same considerations can be applied for the contribution due to sample geometry and size. We only consider a horizontal cut through the otherwise cylindrical sample normally used for diffraction measurements. This leaves us with a circular cross section for which the FWHM of a Gaussian distribution is calculated as *w*_Sample_ = 4.33 mm for a sample with a diameter of *d* = 6 mm.

The uncertainty caused by the divergence is mainly governed by the horizontal divergence. Monte Carlo simulations of the neutron guide system employing the program package *VITESS* (Wechsler *et al.*, 2000 ▸; Lieutenant *et al.*, 2014 ▸) suggest that the horizontal divergence of Δ2θ_Divergence_ = 0.11° = 1.92 × 10^−3^ rad shows almost no dependence on the wavelength.

Finally, POWGEN’s detector panels are arranged around the sample following an equi-angular spiral with
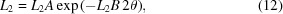
where *L*_2_ is the radial distance and 2θ is the scattering angle in radians. *L*_2_ *A* and *L*_2_ *B* are arbitrary constants describing the size and the curvature of the spiral, respectively. In accordance with previous data (Huq *et al.*, 2010 ▸), initial design values of *L*_2_ *A* = 4.7 m and *L*_2_ *B* = 0.1 rad^−1^ were chosen. A fit using equation (12) to the real distances of the detector pixels as extracted from the detector information provided with each dataset yields *L*_2_ *A* = 4.64 m and *L*_2_ *B* = 0.24 rad^−1^.

**Figure 5:**
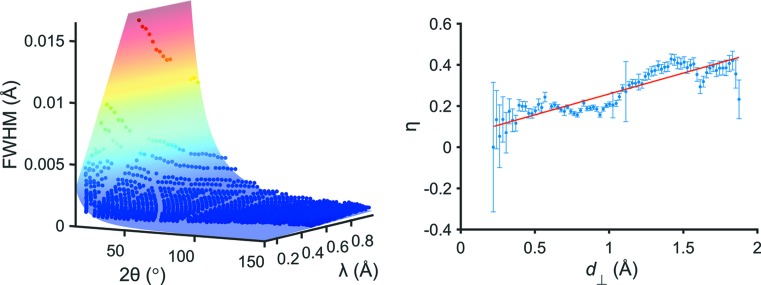
(Left) Extracted FWHM values (colored circles) for the neutron powder diffraction dataset of diamond using binning sizes of 80 *d*_⊥_ slices and Δ_Div_ = 10. The transparent surface depicts the deduced analytical function. (Right) Extracted values for the Lorentzian mixing parameter η together with a linear fit. Error bars denote the 95% confidence interval.

Thus, the complete angular resolution is estimated as 
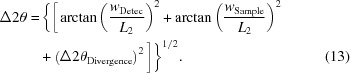
The time uncertainty and pulse duration is essentially determined by the characteristics of the poisoned decoupled moderator, and in a first approximation is proportional to the neutron wavelength. With an estimate of Δ*t* = 10 µs and a total time of flight of *t* = 16.05 ms for a flight path length of *L* = 63.5 m and for a wavelength of λ = 1.0 Å, one obtains
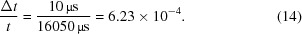
The uncertainty in length Δ*L* with respect to the neutron guide, the sample and the detector has been calculated by *VITESS* simulations. By estimating the total flight path *L* as the mean traveling distance of 63.5 m and setting Δ*L* = 2 mm (FWHM), equation (12) becomes
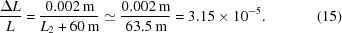
The final instrumental resolution emerges by inserting equations (13), (14) and (15) into equation (10). A summary of all parameters contributing to the calculation of *H* together with their initial values is given in Table 5 ▸. Additionally, in the left part of Fig. 5 ▸ the extracted *H* values are shown together with the hyper-surface of the analytical model. Even though none of the parameters deduced above were refined, there is already an excellent agreement of the FWHM values with an overall *R* ^2^ = 0.957. For refining the analytical function three parameters (*u*_1_, *u*_2_ and *u*_3_) as multipliers to the Δθ, Δ*t* and Δ*L* values can be used, but it is unsurprising that especially *u*_2_ and *u*_3_ exhibit a strong correlation as Δ*t* and Δ*L* have rather similar contributions to the instrumental resolution. It seems therefore convenient to just keep *u*_1_ and rather add an additional resolution term Δ_add_, which comprises all unknown additional contributions: 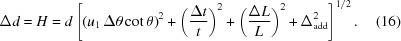
Refining these two parameters yields *u*_1_ = 0.886 (5) and Δ_add_ = 0.00135 (3), with an even better overall *R* ^2^ of 0.987. This parameterization has two main advantages:

(1) The behavior of the FWHM for all reflections observed in the angular- and wavelength-dispersive diffraction pattern is based on well defined physical and geometrical properties of the instrument and the sample.

(2) With deviations from instrumental resolution, properties of the sample can be revealed. For example, micro-strain and/or size effects can be analyzed by an additional contribution to the FWHM.

To further stabilize the parameterization, all reflections in a single *d*_⊥_ slice were refined using the same η value. The extracted values indicate an increase with larger *d*_⊥_ values, illustrating the different contributions of the resolution terms in different parts of the two-dimensional pattern. A linear fit approximation 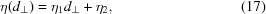
with the data shown in the right part of Fig. 5 ▸, yields initial values of η_1_ = 0.2028 Å^−1^ and η_2_ = 0.05559.

**Figure 6:**
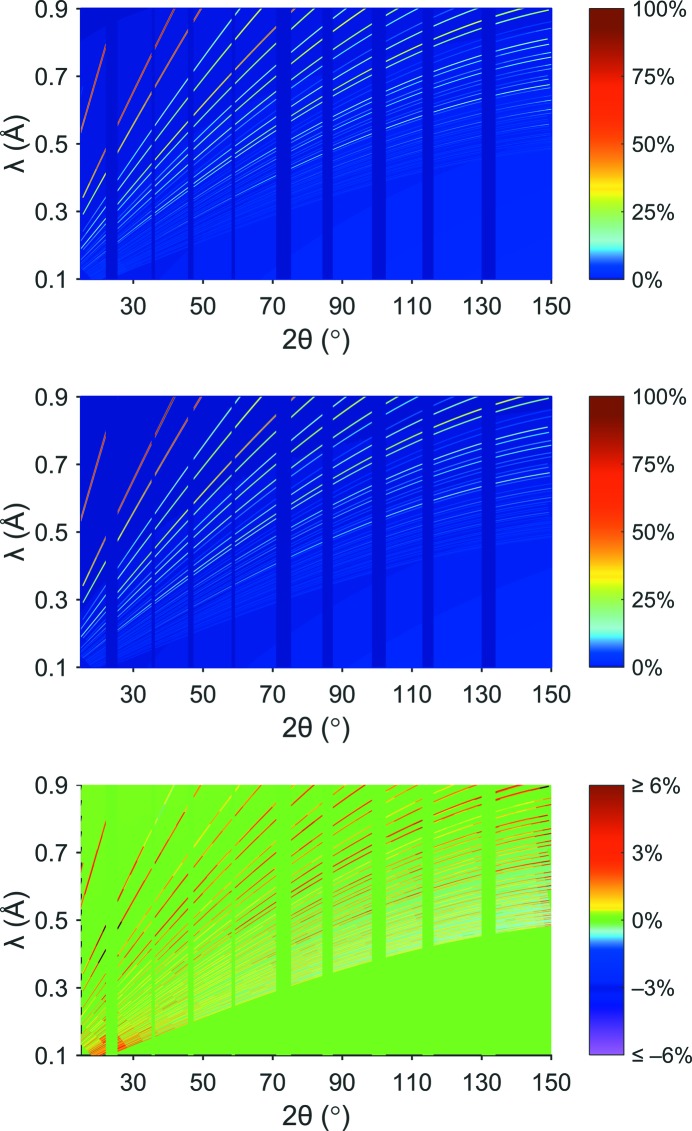
Measured (top), fitted (middle, *R* ^2^ = 0.993) and differential (bottom) diffraction patterns of diamond data from POWGEN with 40 *d*_⊥_ slices and Δ_Div_ = 10. The color bar denotes the intensity as a percentage of the largest intensity peak in the experimental diffraction pattern. Reflections have been drawn broader than they really are to enhance the overall clarity.

The last step is to apply the approach laid out in the preceding sections to the entire diffraction pattern – and thus perform a Rietveld refinement. As has already been pointed out (Jacobs *et al.*, 2015 ▸), at the current stage all structural parts contributing to the intensity calculation are handled by supplying the *h*, *k*, *l*, *M* and *F* ^2^ values derived from the conventional (one-dimensional) Rietveld refinement using the *FullProf* program package (Rodriguez-Carvajal, 1997 ▸) to the fitting routines written in *MATLAB* (http://www.mathworks.com). The results of this refinement are summarized in Table 6 ▸ and shown in Fig. 6 ▸. As is evident from Fig. 6 ▸ the calculated pattern is in very good agreement with the experimental one, with an overall *R* ^2^ of 0.993 and a profile *R* value of *R*_P_ = 9.99. Compared to the one-dimensional case, the *R*_P_ value is slightly larger owing to the somewhat enhanced noise in the two-dimensional pattern. The lattice parameter *a* derived from the two-dimensional refinement is slightly higher than that from the standard Rietveld refinement using *FullProf*. This is expected as in the two-dimensional case only a standard pseudo-Voigt function was used, whereas in the one-dimensional case the pV function with back-to-back exponentials was employed.

**Figure 7:**
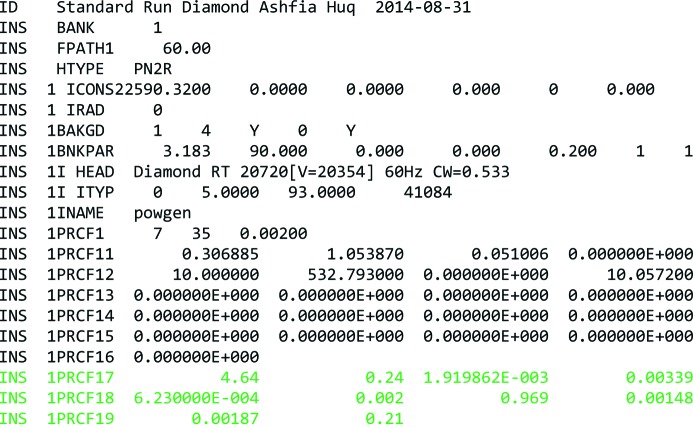
An example of an IRF for the POWGEN instrument, based on *GSAS* input format. Newly added parameters are shown in green.

Given the results from the final refinement of the complete diffraction pattern, we may now compile a two-dimensional instrumental resolution file as is provided by all modern TOF instruments assuming that the measured sample, apart from its volume, has no influence on the peak profile. An example of such an IRF input for *GSAS* is presented in Fig. 7 ▸. The format shown is based on the most recent *GSAS* parameter file provided on the POWGEN home page for cycle 2014-B (PGHR_60-2014B.prm). Using a two-dimensional version of the pV function with back-to-back exponentials (profile function 7) some important keywords were changed. First, ‘HTYPE’ was changed from ‘PNTR’ to ‘PN2R’ to indicate a two-dimensional refinement of powder neutron data. Second, ‘PRCF’ for the profile function type was changed from 3 (one-dimensional, pV-b2b) to 7 (two-dimensional, pV-b2b). Function 6 will use a two-dimensional pV function. Additional parameters are appended to the standard IRF using the sections for PRCF17, PRCF18 and PRCF19. Those lines hold the parameters introduced above. Of course, this file is a rather simplified version of such an input file for the two-dimensional approach as it is not yet available in any of the distributed software packages. It will thus have to be changed according to the needs of the respective refinement software.

From a more general perspective, we also mention that the *d*–*d*_⊥_ binning is perfectly suited for nonlinear multi-bank approaches, which have been used to analyze data from POLARIS and GEM (Williams *et al.*, 1997 ▸; Hull *et al.*, 1992 ▸), as each *d*_⊥_ slice can be regarded as a single pattern that can be fed into current refinement programs. Nevertheless, even in this case the parameters describing the peak profile of each slice have to be linked according to the two-dimensional approach laid out in this work. The limiting case of a single *d*_⊥_ bin is, in fact, the standard ‘diffraction focusing’ approach currently applied by almost every TOF instrument. We reiterate that by reducing the number of *d*_⊥_ slices the original information content as well as the pristine instrumental resolution is lost. As has been shown above, however, refining angular- and wavelength-dispersive data is possible without prior data reduction according to the two-dimensional approach presented here.

## 4. Conclusion and outlook   

We have elaborated on the Rietveld refinement of angular- and wavelength-dispersive datasets with special attention to the binning scheme, which has a huge impact on the reliability and interpretability of diffraction patterns. We chose a standard diamond sample obtained from the POWGEN instrument to determine a two-dimensional instrument resolution function using a fundamental description and values. We extended our approach of using the *d*–*d*_⊥_ coordinate system by also including the transformations back to 2θ/λ or any other coordinates allowing us to most efficiently describe an instrumental or sample behavior. For the description of the profile function, *i.e.* the FWHM and shape parameterization, the *d*–*d*_⊥_ system is a good choice. This naturally follows from the definition of *d*_⊥_, which is the solution of a differential equation requiring orthogonal cuts to be exactly perpendicular to all reflections. This is not the case in other possible coordinate systems like *d*, 2θ or *Q*, 2θ. This finding allows a much easier data representation and binning procedure.

In particular, regarding the binning we have chosen a logarithmic binning in *d* spacing and a linear binning in *d*_⊥_, and we compare it with the previously chosen 2θ and λ binning. The advantage of the *d*–*d*_⊥_ coordinate system can be further exploited by using a resolution-derived and thus varying *d* and *d*_⊥_ binning to achieve an optimized data-point distribution with minimal sacrifice of information.

Although the future POWTEX instrument (which features a large detector coverage and cost-efficient detector design) as well as the DREAM instrument at the ESS will greatly benefit from the two-dimensional approach, we have demonstrated a major gain regarding the right description of the instrumental resolution even for the POWGEN instrument. This is especially true as the instrumental contributions are now based on instrumental design parameters and sample geometry, reflecting real properties of the experimental setup. Additional contributions from the sample can now be addressed more efficiently and attributed to the different terms of the resolution function according to their origin type. In response to similar procedures at existing instruments, we have also derived an instrumental profile function for POWGEN which may be provided to the user in the form of an instrumental resolution file.

In addition, the new approach will give experienced TOF users the opportunity to check the data quality more thoroughly. Furthermore, we believe that the novel approach allows more freedom to utilize less complex detector arrangements. For example, the geometry chosen for the POWTEX instrument is cylindrical, with its axis in the beam direction, adapting to the Debye–Scherrer cones. However, the proposed approach should be of general interest for powder diffraction instruments at pulsed neutron sources.

With the introduction of two-dimensional profile functions and their specifications given in an instrumental resolution file, future work will aim at providing a better incorporation of sample effects (*e.g.* preferred orientation, absorption and so on). Finally, we aim to implement the multi-dimensional refinement procedure into common Rietveld program suites in order to offer all necessary data-treatment possibilities to the users. This new development is open for joint activities with program authors of existing user software, and we welcome current considerations towards an implementation into *GSAS-II* (Toby & Von Dreele, 2013 ▸). Finally, it is worth noting that for future users a benchmarking of the new methods has to be carried out on refined sample parameters like bond distances or atomic displacement parameters. In order to have a fair comparison, an implementation in a regular software package is what one should aim for.

## Figures and Tables

**Table 1:** Crystallographic data of diamond

	Diamond (Bragg & Bragg, 1913 ▸)	
Lattice parameter (Å)	*a* = 3.55	
Space group	*Fd* 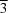 *m* (No. 227)	
Formula units	*Z* = 8	
Atomic sites	C (8*a*)	0 | 0 | 0
Temperature (K)	*T* = 293	

**Table 2:** Comparison of *H* values for selected reflections extracted from datasets obtained with different bin sizes for 2θ and λ using data points in the interval 0.73 ≤ *d*_⊥_ < 0.77 Å

Binning		*d* = 2.059 Å		*d* = 1.076 Å		*d* = 0.477 Å	
Δ2θ (°)	Δλ (Å)	No. / FWHM	Δ*d* (Å) × 10^−2^	No. / FWHM	Δ*d* (Å) × 10^−2^	No. / FWHM	Δ*d* (Å) × 10^−2^
0.05	0.0005	253	1.28 (8)	201	0.37 (2)	112	0.067 (7)

0.1	0.001	51.2	1.44 (1)	52.9	0.42 (3)	28.4	0.09 (2)
0.2	0.001	25.3	2.0 (2)	26.7	0.56 (4)	14.1	0.10 (3)
0.4	0.001	13.2	3.6 (6)	12.8	1.0 (2)	7.74	0.11 (3)

0.1	0.002	25.8	1.5 (2)	26.8	0.47 (5)	14.7	0.15 (3)
0.2	0.002	12.7	2.0 (2)	13.5	0.58 (9)	7.43	0.14 (4)
0.4	0.002	6.84	3.6 (9)	6.66	1.0 (2)	4.07	0.14 (5)

0.1	0.004	13.6	1.7 (3)	13.3	0.6 (1)	7.62	–
0.2	0.004	6.69	2.1 (4)	6.78	0.7 (1)	4.04	–
0.4	0.004	3.46	3.7 (9)	3.43	1.1 (3)	2.09	–

**Table 3:** Comparison of *H* values for selected reflections extracted from datasets obtained using different bin sizes for *d* and *d*_⊥_ for a *d*_⊥_ slice at *d*_⊥_ = 0.75 Å

Binning		*d* = 2.059 Å		*d* = 1.076 Å		*d* = 0.477 Å	
No. of *d*_⊥_ slices	Δ	No. / FWHM	Δ*d* (Å) × 10^−2^	No. / FWHM	Δ*d* (Å) × 10^−2^	No. / FWHM	Δ*d* (Å) × 10^−2^
200	0.0004	15.1	1.24 (5)	8.83	0.37 (1)	5.32	0.07 (1)
80	0.0004	15.1	1.28 (3)	8.83	0.377 (8)	5.32	0.07 (1)
40	0.0004	15.1	1.26 (2)	8.83	0.369 (7)	5.32	0.07 (1)

200	0.0008	7.72	1.24 (6)	4.55	0.37 (1)	2.78	0.07 (2)
80	0.0008	7.72	1.28 (5)	4.55	0.38 (1)	2.78	0.07 (2)
40	0.0008	7.72	1.26 (2)	4.55	0.38 (1)	2.78	0.07 (2)

200	0.0016	3.98	1.2 (1)	2.42	0.39 (2)	1.30	0.06 (3)
80	0.0016	3.98	1.30 (5)	2.42	0.40 (2)	1.30	0.07 (2)
40	0.0016	3.98	1.27 (3)	2.42	0.39 (2)	1.30	0.07 (3)

**Table 4:** Comparison of *H* values for selected reflections extracted from datasets obtained using the resolution-adapted binning scheme for a *d*_⊥_ slice at *d*_⊥_ = 0.75 Å

Binning		*d* = 2.059 Å		*d* = 1.076 Å		*d* = 0.477 Å	
No. of *d*_⊥_ slices	Δ_Div_	No. / FWHM	Δ*d* (Å) × 10^−2^	No. / FWHM	Δ*d* (Å) × 10^−2^	No. / FWHM	Δ*d* (Å) × 10^−2^
80	5	5.23	1.27 (3)	5.04	0.38 (2)	4.85	0.07 (1)
40	5	5.30	1.26 (3)	5.09	0.372 (8)	4.89	0.07 (1)
20	5	5.16	1.31 (3)	4.98	0.39 (1)	4.81	0.07 (1)
10	5	5.43	1.27 (2)	5.22	0.37 (1)	4.97	0.07 (1)
80	10	10.3	1.26 (3)	10.3	0.38 (1)	9.98	0.070 (7)
40	10	10.4	1.26 (2)	10.1	0.369 (7)	10.0	0.070 (7)
20	10	10.1	1.30 (2)	9.90	0.383 (8)	9.83	0.072 (7)
10	10	10.6	1.26 (2)	10.2	0.362 (6)	9.70	0.066 (6)

**Table 5:** Parameters for the instrumental resolution function and the Lorentzian mixing parameter together with their initial values

Parameter	Initial value
*w*_Detec_ (m)	3.39 × 10^−3^
*w*_Sample_ (m)	4.33 × 10^−3^
Δ2θ_Divergence_	0.11° = 1.92 × 10^−3^ rad
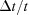	6.23 × 10^−4^
Δ*L* (m)	2.00 × 10^−3^
*L*_2_ *A* (m)	4.64
*L*_2_ *B* (rad^−1^)	0.24
η_1_ (Å^−1^)	0.2028
η_2_	0.05559

**Table 6:** Results of the two-dimensional pattern fitting using MATLAB for the experimental data of diamond (POWGEN) and comparison with the standard one-dimensional refinement using *FullProf*

	Two-dimensional	*FullProf*
No. of parameters	5	7
No. of data points	∼154000	4730
No. of reflections	96	95
Calculation time (s)†	∼210	∼30
Lattice parameter (Å)	3.56788 (7)	3.56745 (4)
Background	Manually subtracted and smoothed	
*B*_iso_	0.261 (3)	0.115 (8)
*S*	0.00369 (2)	Not comparable
*u*_1_	0.895 (3)	
Δ_add_	0.00120 (1)	
*R*_p_	9.99	9.6

† ASUS K73S Notebook with Intel Core i5-2410M (2 Cores @ 2.3 GHz) and 6 GB of RAM.
